# Global research status and frontiers on microvascular invasion of hepatocellular carcinoma: A bibliometric and visualized analysis

**DOI:** 10.3389/fonc.2022.1037145

**Published:** 2022-12-14

**Authors:** Tao He, Jieyu Zou, Ke Sun, Juan Yang, Tingting Lei, Lin Xu, Jinheng Liu, Sineng Yin, Guangkuo Li

**Affiliations:** ^1^ Department of Hepatobiliary Surgery, Chengdu Second People’s Hospital, Chengdu, Sichuan, China; ^2^ Depatment of Oncology, The Second Affiliated Hospital of Chongqing Medical University, Chongqing, China

**Keywords:** microvascular invasion, bibliometric analysis, prognosis, treatment, hepatocellular carcinoma

## Abstract

**Introduction:**

Over the past decade, several studies on the microvascular invasion (MVI) of hepatocellular carcinoma (HCC) have been published. However, they have not quantitatively analyzed the remarkable impact of MVI. Therefore, a more comprehensive understanding of the field is now needed. This study aims to analyze the evolution of HCC-MVI research and to systematically evaluate the scientific outputs using bibliometric citation analysis.

**Methods:**

A systematic search was conducted on the Web of Science Core Collection on 2 May 2022 to retrieve studies on HCC-MVI published between 2013 and 2022. Then, a bibliometric analysis of the publications was performed using CiteSpace, VOSviewer, and other visualization tools.

**Results:**

A total of 1,208 articles on HCC MVI were identified. Of these, China (n = 518) was the most prolific country, and Fudan University (n = 90) was the most notable institution. Furthermore, we observed that Lau Wan Yee participated in most studies (n = 26), and *Frontiers in Oncology* (IF2020:6.24) published the highest number of documents (n = 49) on this subject, with 138 publications. The paper “Bray F, 2018, CA-CANCER J CLIN, V68, P394” has the highest number of co-cited references, with 119 citations. In addition, the top three keywords were “survival”, “recurrence”, and “microvascular invasion”. Moreover, the research hot spots and frontiers of HCC-MVI for the last 3 years included imaging characteristics and transarterial chemoembolization (TACE) therapy studies.

**Conclusions:**

This study comprehensively summarized the most significant HCC-MVI documents from past literature and highlighted key contributions made to the advancement of this subject and the advancement of this field over the past decade. The trend of MVI research will gradually shift from risk factors and prognosis studies to imaging characteristics and TACE therapy studies.

## Introduction

As the sixth incident and the fourth leading cause of cancer death worldwide, the number of new annual cases and deaths from primary liver cancer is now approximately is 841,000 and 782,000 per year around the world, respectively (1). Hepatocellular carcinoma (HCC) is the most common pathological type of liver cancer, accounting for 90% of all types in China (2). Microvascular invasion (MVI) refers to the presence of clusters of cancer cells in blood vessels with endothelial cell linings, most commonly pronounced in the branches of the paraneoplastic portal vein (including the intra-capsular blood vessels) under the microscope, which is an aggressive pathological feature of HCC (2). Moreover, the presence of MVI has been reported to be a poor prognosis factor for recurrence and long-term survival after liver resection or transplantation (3).

Bibliometric analysis is a quantitative tool, which combines statistical methods with information visualization technology to identify core entities and developmental trends, focusing on specific subjects or research domains. Thus, the technique could offer opportunities to improve the timeliness, accessibility, and reproducibility of literature-based studies during research. Therefore, this method has recently been widely employed in biomedicine and mechanical engineering (4–6). Specifically, CiteSpace, VOSviewer, and R software are the most commonly employed statistical quantitative analysis tools.

Unfortunately, only a few studies have analyzed these published documents using bibliometric citation analysis (4, 7, 8). Xu et al. (7) used VOSviewer to analyze the 100 most influential articles over the past four decades, and the R software was used to analyze the evolution of HCC treatment. Yang et al. (8) completed a bibliometric analysis on HCC magnetic resonance imaging research. However, these manuscripts only showed that MVI is the research hot spot of HCC and needed further discussion. Considering the research gaps mentioned above, the study addressed the following research question: “Can we establish a comprehensive bibliometric analysis of HCC-MVI and guide clinicians to adopt it in their work?”. Therefore, this study tried to perform a comprehensive and multidirectional quantitative analysis to fill the deficiency in this domain. Subsequently, our ultimate objectives aimed to provide clinicians and scholars with information that will develop a general understanding of this evolution, facilitating a meaningful insight into future directions relative to advances in the HCC-MVI field.

## Materials and methods

### Literature search and screening

**Figure 1:**
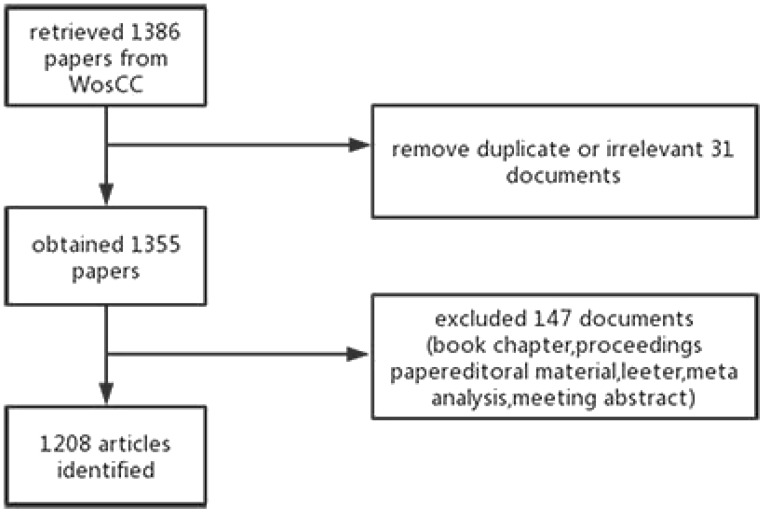
A flowchart of the study to retrieve HCC-MVI articles from the WosCC database.

This research quantificationally evaluated the existing scientific outputs to characterize the evolution of HCC-MVI research over the past decade. A literature search was conducted on 2 May 2022 to retrieve published literature from 2013 to 2022 from the Science Citation Index-Expanded (SCIE) of the Web of Science Core Collection (WoSCC), which is an appropriate search engine containing primary citation sources and publishes peer-reviewed journals and conferences. We conducted all searches on the same day to avoid database update bias, using the following search key terms: Topic = (“primary hepatic cancer”) OR (“primary liver cancer”) OR (“hepatocellular carcinoma”) OR (“malignant hepatoma”) OR (“primary liver carcinoma”) OR (“primary hepatic carcinoma”) AND (“microvascular invasion”). The types of publications were limited to “article” and “review” to ensure the representativeness of the included studies. Meanwhile, this research only included published documents whose language was English. Two reviewers (JZ and KS) independently identified the raw data, downloaded articles from WoSCC, and excluded duplicate/irrelevant documents. In case of a discrepancy between the two reviewers, a consensus was reached with the help of a third independent reviewer (TH). A total of 1,208 most relevant documents on HCC-MVI are finally collected. The study flowchart is shown in **Figure 1**.

### Statistical analysis and visualization

The number of annual publications was imported into Excel 2019, and the trend was further analyzed. Subsequently, the CiteSpace (version 5.8 R3) was adopted as the search tool to analyze institutions, co-cited references, and keywords. Time slices were set to 1 year per slice, and selection criteria were selected as g-index. In the visual network maps, node colors change from cold to warm from the inside to the outside, representing the year from the original to the most recent. Pruning: network pruning method. Modularity Q (Q-value): Clustering module value, it is generally assumed that Q > 0.3 means that the clustering structure is significant. Weighted mean silhouette S (S-value): The average silhouette value of the cluster, it is generally considered that S > 0.5 clustering class is reasonable, and S > 0.7 means that the clustering is convincing. Analysis of authors, co-cited authors, and journals was performed using VOSviewer (version 1.6.18). In addition, a world map of the publications’ number and countries’ cooperation was created using ECharts (version 4.5.0) and SCImago Graphica Beta (version 1.0.18), respectively. Furthermore, the visual knowledge graph consisted of nodes and linear connections. Each node in the graph represented a key point, and the node’s size represented the frequency of occurrence and citation.

## Results

Our investigation revealed that 6,799 authors who completed 1,208 manuscripts in this study were from 1,088 institutions in 41 countries, of which articles and reviews were 1,101 and 107, respectively. Moreover, they were published in 297 journals, citing 22,505 references from 2,673 journals.

### Trends in publications

**Figure 2:**
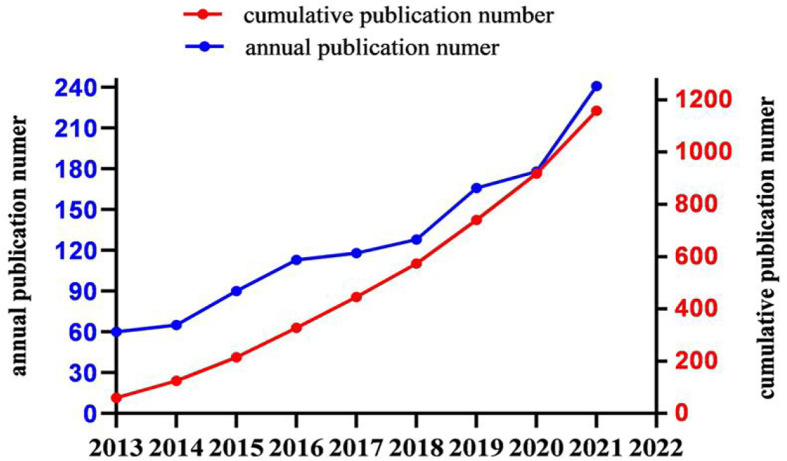
Number of publications by year (Y = 1E−302e^0.3476x^ and R^2^ = 0.9503, where Y is the cumulative publicaton number and x is the published year).

The number of studies published in each year can help us understand the general trends in the relevant research. The annual publications showed an obvious upward trend during the period under review (**Figure 2**). According to **Figure 2**, the equation of prediction between cumulative publicaton number (Y) and published year (x) is Y = 1E−302e^0.3476x^ and can predict the documents published yearly having an R-square of 95% (R^2^ = 0.9503). Moreover, the annual output number has markedly increased yearly since 2015, indicating that MVI has received high attention in this period. Furthermore, investigations revealed that the average annual output was approximately 120 documents, with 2,021 being the year with the most publications.

### Distribution of countries and institutions

**Figure 3:**
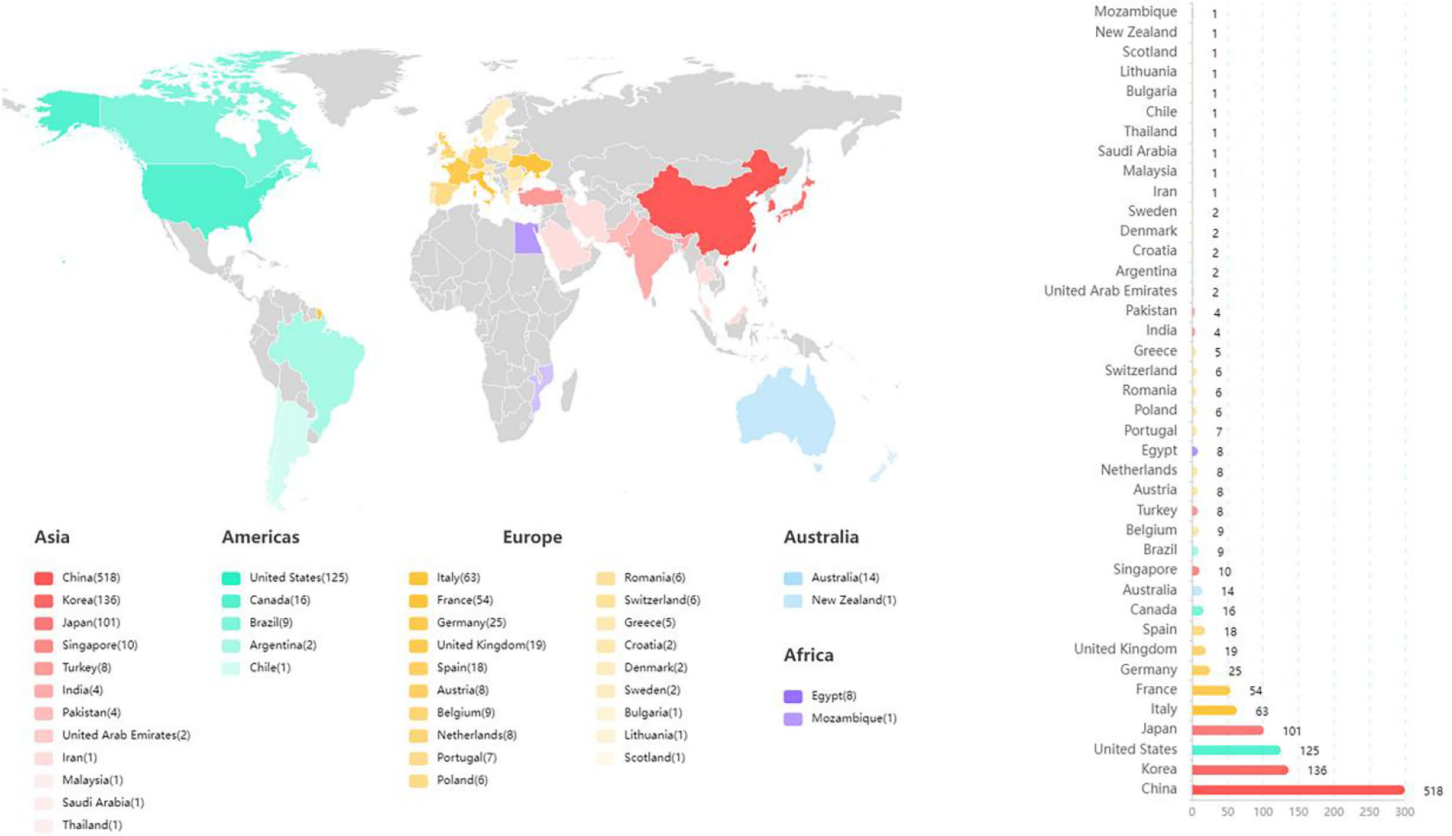
Number of publications by country.

**Figure 4:**
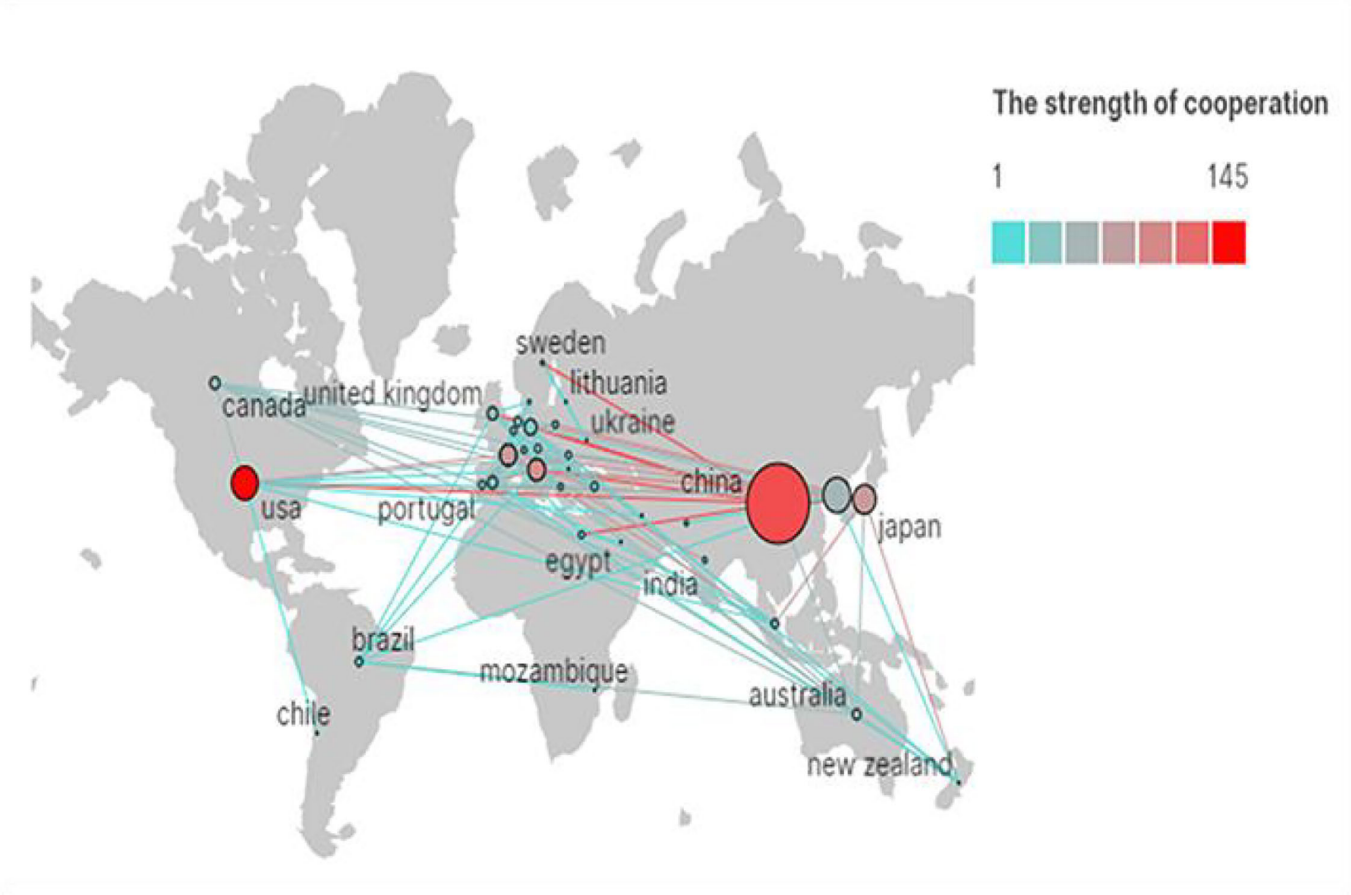
The network map of countries cooperation (the size of the node represents the number of publications, and the color of the line shows the strength of cooperation).

**Figure 5:**
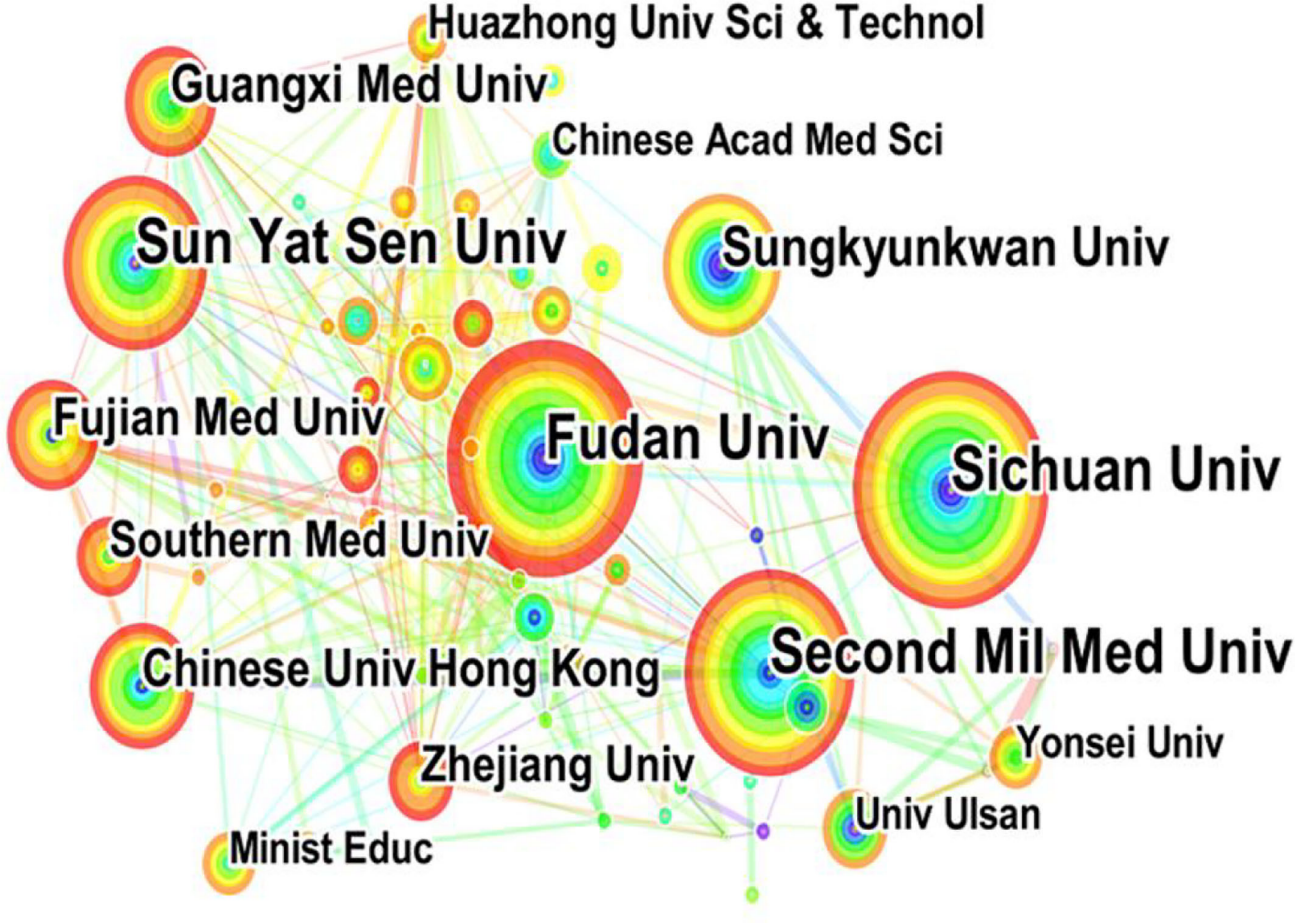
The bibliometric analysis of active institutions in the HCC-MVI research (the lines represent cooperation relationships, and the colors in the nodes represent the years).

Among the 41 countries of the manuscripts identified (**Figure 3**), China (including Hong Kong, Macau, and Taiwan) participated in most studies (n = 518), accounting for 42.88% of all documents, followed by South Korea (136), the US (125), and Japan (101), which were significantly higher than those of other countries. **Figure 4** depicts the partnerships among countries that published these articles, demonstrating cooperation among the various countries. Notably, China had the strongest cooperative relations with the US in this field. Meanwhile, 1,088 institutions contributed to MVI research. **Figure 5** shows the partnerships between the institutions actively conducting research. The top three institutions that published the most articles included Fudan University (90 documents), the Second Military Medical University (83 documents), and the Sichuan University (78 documents), with 1,442, 1,708, and 1,139 total citations (TCs), respectively (**Table 1**). However, the Chinese University of Hong Kong ranked first based on average citations (n = 30.35).

**Table 1:** The top 10 institutions contributed to publications in the HCC-MVI research.

Rank	Institution	Country	Counts	TCs	Average TCs
1	Fudan University	China	90	1,442	16.02
2	Second Military Medical University	China	83	1,708	20.57
3	Sichuan University	China	78	1,139	14.60
4	Sun Yat Sen University	China	74	1,065	14.39
5	Sungkyunkwan University	Korea	49	883	18.02
6	The Chinese University of Hong Kong	China	37	1,123	30.35
7	Fujian Medical University	China	36	273	7.58
8	Guangxi Medical University	China	35	434	12.40
9	Southern Medical University	China	29	455	15.68
10	Zhejiang University	China	28	310	11.07

Average TCs: the ratio of TCs to publication counts.

### Analysis of authors and co-cited authors

**Figure 6:**
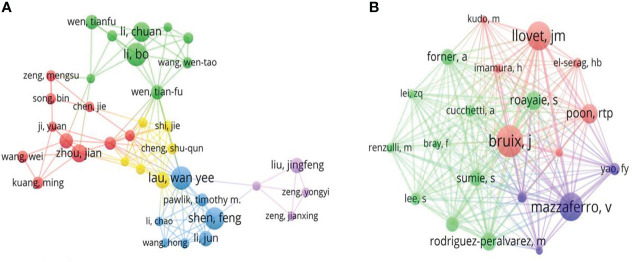
The knowledge map of active authors and co-cited authors in the HCC-MVI research. The node size represents the frequency of authors. **(A)** Active authors. **(B)** Active co-cited authors.

With more than 6,000 authors, the knowledge map provided the information on the most influential authors including their collaborative relationships (**Figure 6**). Among the top 10 contributive authors (**Table 2**), Lau Wan Yee and Li Bo were the most productive authors, each with 26 publications, followed by Shen Feng with 22 publications. As a co-cited author, Bruix J (499 citations) ranked first, followed by Llovet JM (454 citations) and Mazzaferro V (443 citations).

**Table 2:** The top 10 authors and co-cited authors in the HCC-MVI research.

Rank	Author	Counts	Co-cited author	Counts
1	Lau Wan Yee	26	Bruix J	499
2	Li Bo	26	Llovet jm	454
3	Shen Feng	25	Mazzaferro V	443
4	Li Chuan	22	Poon rtp	304
5	Joh Jae-Won	22	Roayaie S	295
6	Kim Jong Man	21	Rodriguez-peralvarez m	276
7	Zhou Jian	20	Forner A	246
8	Wu Mengchao	18	Sumie S	243
9	Fan Jia	17	Lim KC	233
10	Li Jun	17	European assoc study liver	207

### Analysis of active journals

**Figure 7:**
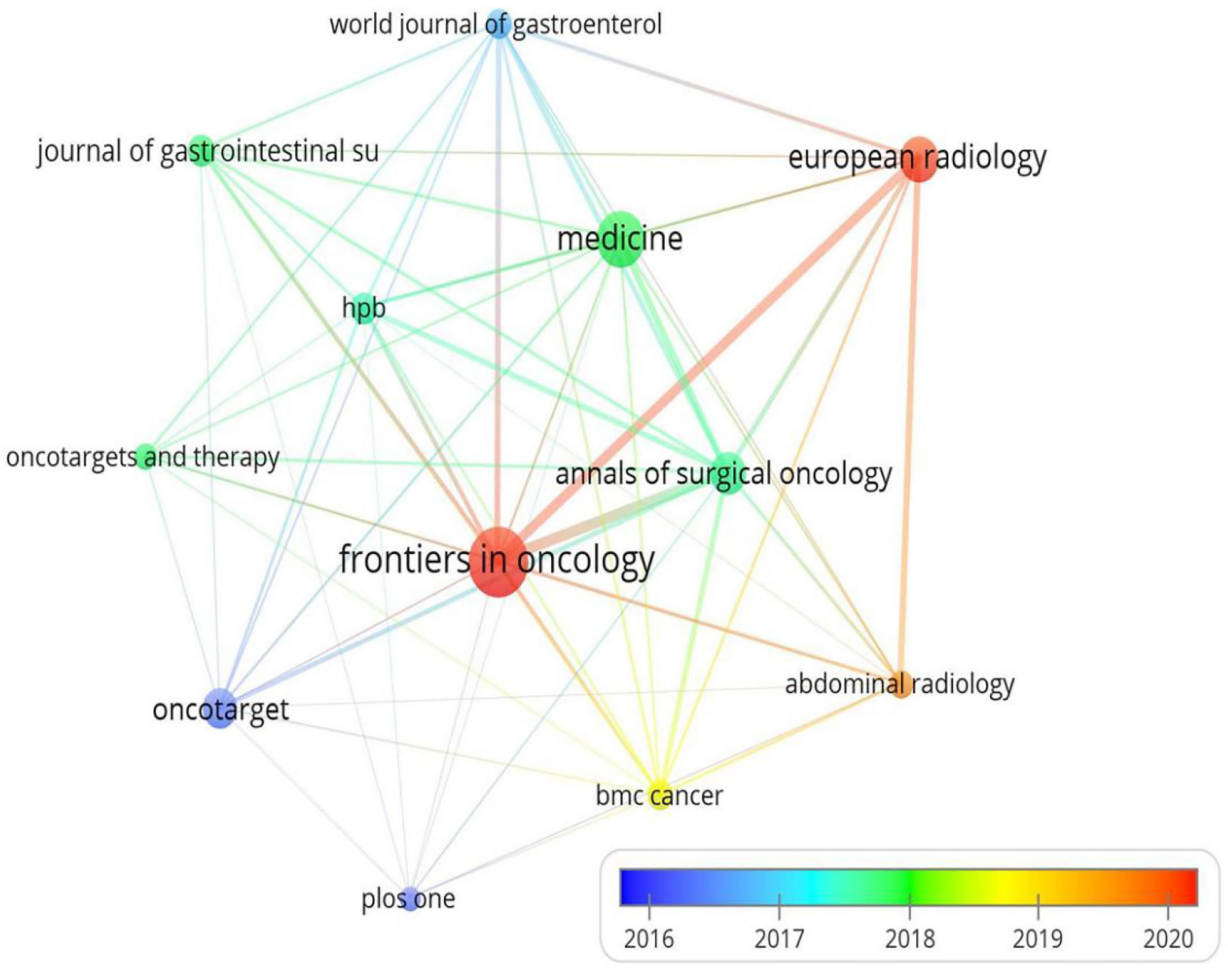
The bibliometric analysis of active journals in the HCC-MVI research (the colors in the nodes represent the years).

**Figure 8:**
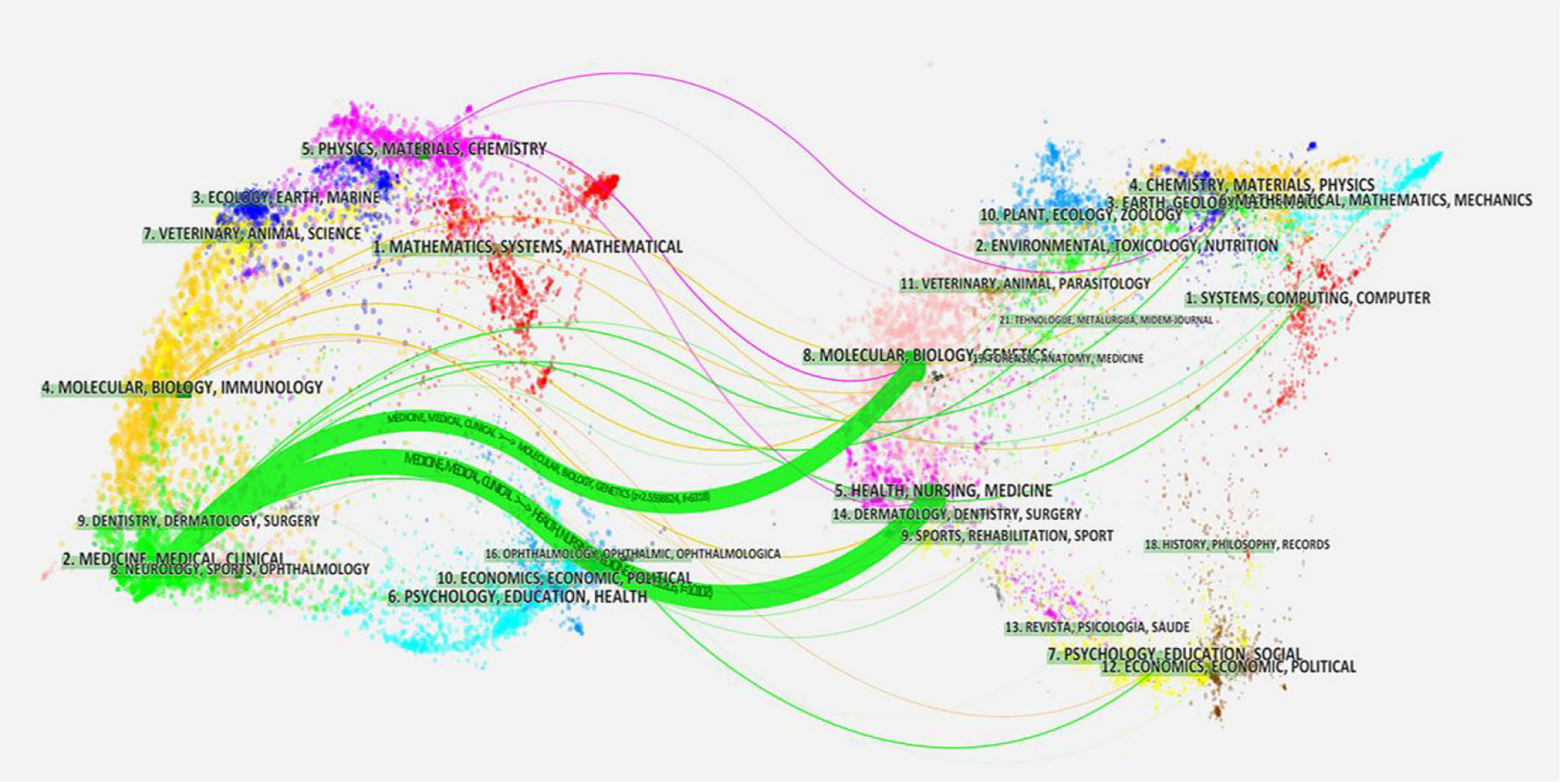
The dual map overlay of journals in the HCC-MVI field (left, citing journals; right, cited journals).

The results show that 297 journals participated in this field. **Table 3** lists the number of articles published, the top 10 journals, their TCs, impact factors (IFs) 2020, and Journal Citation Report (JCR) areas. Accordingly, *Frontiers in Oncology* published 49 documents and ranked first. However, *Annals of Surgical Oncology* was the journal with the highest number of citations (1,014 TCs). Furthermore, the IF of journals, excluding *Oncotarget*, ranked from 1.89 (*Medicine*) to 6.24 (*Frontiers in Oncology*), with an average IF of 4.34. Simultaneously, approximately 70% of the top 10 journals scored Q1/Q2 in the JCR partition. Recently, *Frontiers in Oncology* and *European Radiology* have been the most distinguished journals in the field (**Figure 7**). As shown in **Figure 8**, the two green citation paths indicated that journals published in molecular/biology/genetics and health/nursing/medicine were also cited by medicine and medical/clinical journals.

**Table 3:** The top 10 journals that contributed to publications in the HCC-MVI research.

Rank	Journal	Counts	TCs	Average TCs	IF2020	JCR area
1	Frontiers in Oncology	49	138	2.82	6.24	Q2
2	Medicine	40	319	7.98	1.89	Q3
3	European Radiology	32	607	18.97	5.32	Q1
4	Annals of Surgical Oncology	30	1,014	33.80	5.34	Q1
5	Oncotarget	29	670	23.10	NA	NA
6	Journal of Gastrointestinal Surgery	23	332	14.43	3.45	Q2
7	HPB	22	457	20.77	3.65	Q1
8	World Journal of Gastroenterology	21	732	34.86	5.74	Q2
9	Bmc Cancer	21	447	21.29	4.43	Q3
10	Abdominal Radiology	20	217	10.85	3.04	Q2

### Cited references analyses

**Figure 9:**
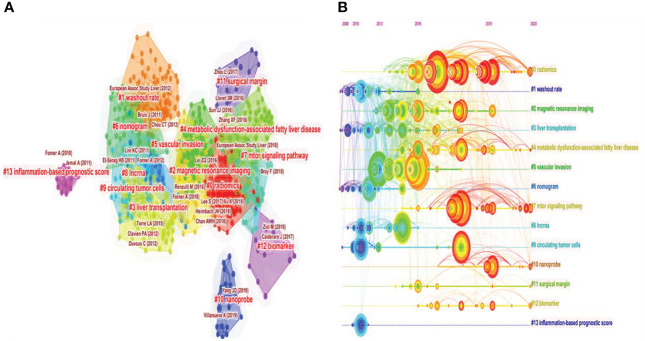
The cluster view and timeline view of co-cited references in the HCC-MVI research. **(A)** Cluster view. **(B)** Timeline view.

The higher the frequency of co-citations means the more vital the publication. We adopted CiteSpace to characterize the relationship between co-cited references based on the retrieved literature. The 14 different clusters made through CiteSpace were shown in **Figure 9A**. The ordinal number of clusters was arranged by cluster size. The smaller the ordinal number, the larger the cluster. Hence, the largest cluster was “#0 radiomics”. Furthermore, we observed that “#0 radiomics” and “#4 metabolic dysfunction–associated fatty liver disease” had developed into the hottest research topics over the past 2 years. **Table 4** shows the analysis of the top 10 co-cited references (**Figure 9B**). Of the 1,208 manuscripts identified, three articles were cited more than 100 times. Moreover, the articles published by Bray F et al. in 2018 ranked first with 119 citations, followed by those by Lei Z, with 117 citations.

**Table 4:** The top 10 co-cited references in the HCC-MVI research.

Rank	Co-cited references	Counts
1	Bray F, 2018, CA-CANCER J CLIN, V68, P394	119
2	Lei Z, 2016, JAMA SURG, V151, P356	117
3	Lee S, 2017, J HEPATOL, V67, P526	114
4	Renzulli M, 2016, RADIOLOGY, V279, P432	96
5	Xu X, 2019, J HEPATOL, V70, P1133	79
6	Torre LA, 2015, CA-CANCER J CLIN, V65, P87	78
7	Rodriguez-Peralvarez M, 2013, ANN SURG ONCOL, V20, P325	74
8	European Assoc Study Liver, 2018, J HEPATOL, V69, P182	71
9	Cong WM, 2016, WORLD J GASTROENTERO, V22, P9279	65
10	Forner A, 2018, LANCET, V391, P1301	63

### Analysis of keywords and burst keywords

**Figure 10:**
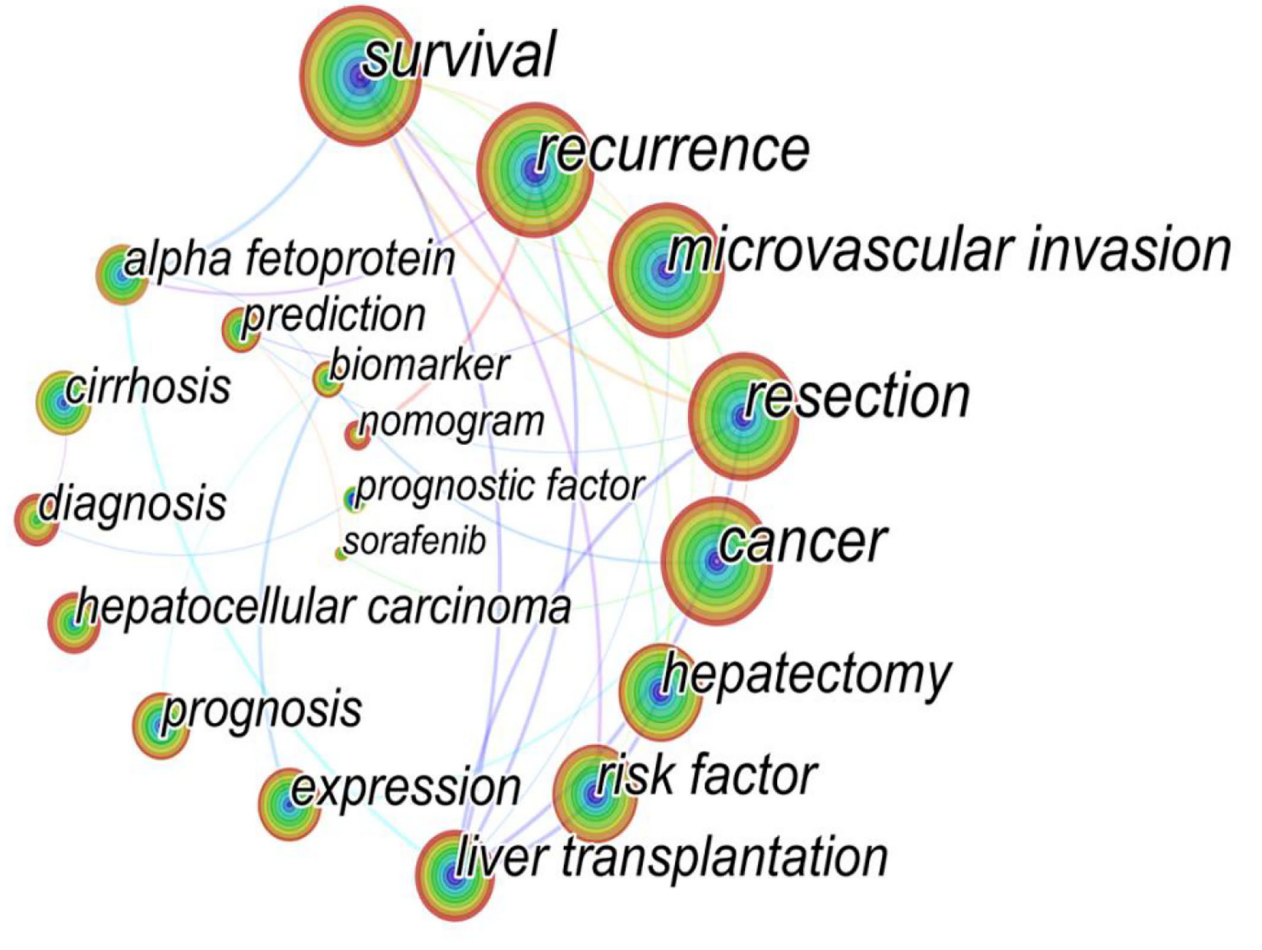
The map of active keywords in the HCC-MVI research (the node size represents the frequency of keyword occurrences).

**Figure 11:**
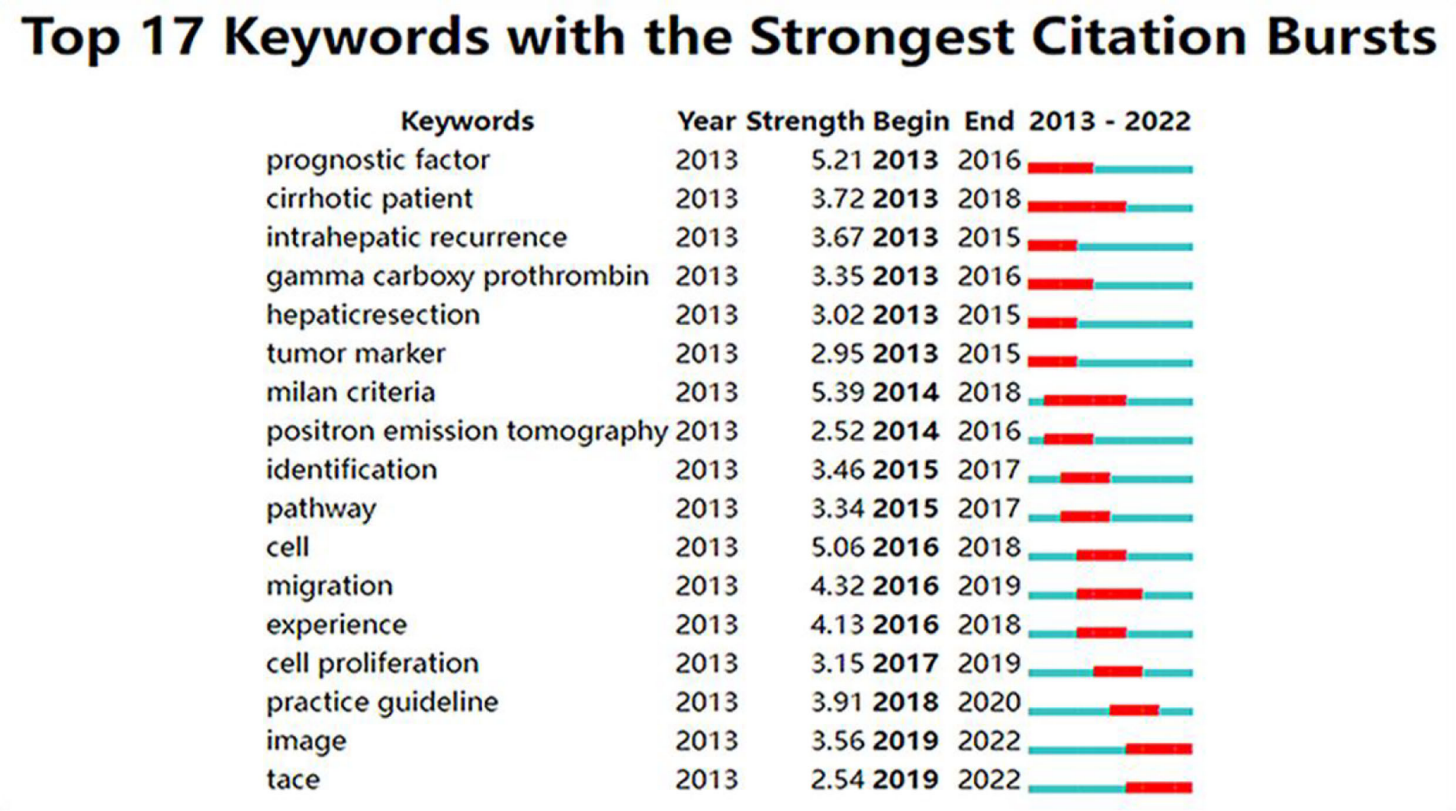
The keywords with the strongest citation bursts from 2013 to 2022 in the HCC-MVI research.

The high-frequency keywords in the article can help us understand the main research hot spots of the field. Therefore, we analyzed the active keywords mentioned most frequently and the timeline view of these studies (**Figure 10**). Statistics data showed “survival” followed by “recurrence” and “microvascular invasion” as the keywords with the highest frequency. Meanwhile, “liver transplantation”, “risk factor”, and “alpha fetoprotein” also occupied the dominating position of keywords. Keywords with higher burst values over a period mean that they have received special attention during the corresponding time intervals and may become a new research trend. **Figure 11** shows that the “Milan criteria” had the strongest citation burst of 5.39. “Tumor marker”, “hepatic resection”, and “prognostic factor” have become hot keywords in early studies. Recently, “image” and “TACE” have become the focus in this field.

## Discussion

Because MVI can only be confirmed by postoperative pathology and existing studies have found that it is closely related to the prognosis of HCC, MVI has become one of the hot spots of HCC research in recent years (2, 3). In this study, we included a total of 1,208 manuscripts on HCC-MVI in the WoSCC database according to the inclusion and exclusion criteria. Subsequently, we used CiteSpace, VOSviewer, and other software to perform a bibliometric and visual analysis of articles on HCC-MVI. We tried to conduct a comprehensive and quantitative analysis to provide an extensive and visual analysis of the literature on HCC-MVI, which may help scholars gain a basic understanding, develop areas of focus, identify trends, and pursue further practice in this field.

### Countries/institutions and their cooperation

From the perspective of countries, the top 10 countries that published articles in this field included three Asian countries, five European countries, and two American countries, accounting for 62.5%, 14.81%, and 11.67% of all documents, respectively. Furthermore, our investigation revealed that more than 40% of the countries had more than 100 publications. China, the only developing country in the top 10, also contributed to more than 40% of documents, indicating its high academic impact on this field. Another remarkable phenomenon was that nine Chinese universities were among the top 10 institutions that contributed to publications, showing the dominant position of Chinese researchers in this field. The incidence rate of HCC in Asia was higher than that in Europe and America (1), which may explain why China had the most prolific research on this topic. In addition to this, although they cooperated extensively with other countries, especially the US, their global cooperation was not strong enough (e.g., with Europe). Therefore, wider and closer national cooperation may require further research.

### Citation information

Co-citation refers to two or more documents being cited by one or more articles at the same time. Highly cited information is generally high-quality research in a field that has had a significant impact on innovation and discovery (9). A survey of author information revealed that all of the top 10 active authors published at least 17 articles. However, although eight of the 10 leading contributors were Chinese, which was consistent with the results of institutional research, none of them were reflected in the top 10 co-cited authors who generated high-quality articles and were more influential. As the co-cited author with the highest number, Bruix J (the Barcelona Clinic Liver Cancer Group, University of Barcelona, Spain) contributed extensively to developing HCC management guidelines (10) and completed numerous of high-quality studies (11, 12). Meanwhile, Llovet JM and Mazzaferro V, also with higher H-index, made notable contributions to the HCC field (13, 14). These outstanding scholars have played a significant role in promoting the development of HCC research.

On the basis of the academic journals (**Table 3**), although the retrieved journals in this field were not high-impact factor journals, *Frontiers in Oncology* had the highest IF and published the highest number of articles. Nevertheless, they received the lowest number of citations in the top 10. This phenomenon revealed that the quality of their publications should be improved. This study also observed that, although the *Annals of Surgical Oncology* published original and educational manuscripts on oncology for surgeons from all specialties in academic and community settings, they merely ranked fourth and then got the highest number of citations. The *Annals of Surgical Oncology* is the official journal of the Society of Surgical Oncology and is published for the Society by Springer, which is one of the world’s largest scientific and technical publishers and also has access to a relatively large number of readers and citations. Thus, we guess that this may be one of the important reasons for the existing differences. In addition, the *World Journal of Gastroenterology* was the journal with the highest average number of TCs. In contrast, *Frontiers in Oncology* was the journal with the lowest average number of TCs. This may be because the former journal has a broader scope of research and received more citations from the corresponding journals in the digestive system. However, since 2020, the number of articles published by *Frontiers in Oncology* and *European Radiology* has increased substantially, which may explain one of the reasons for the change in research trends in this field.

On the basis of co-cited references, Bray F, the head of the International Agency for Research on Cancer, comprehensively described the epidemiological characteristics of malignancy and had the highest number of co-citations in 2018 by one document (1). However, we observed that the Chinese authors published only three documents (**Table 4**), indicating that high-quality research requires the further involvement of Chinese scholars. As a Chinese author with the most cited references, Lei Z published a paper “Nomogram for preoperative estimation of microvascular invasion risk in hepatitis b virus-related hepatocellular carcinoma within the Milan criteria”, which elaborated on how to construct a nomogram using clinical and imaging information and achieved an optimal preoperative prediction of MVI in HBV-related HCC within the Milan criteria (15). What is noteworthy is that the document was only ranked sixth in the top 10 co-cited references, written by an accomplished medical epidemiologist, Torre LA, and published in the journal of *Ca-A Cancer Journal for Clinicians*, with the highest IF of 508 in the academia (16). Simultaneously, although this study was the most cited from a Q1 journal, it was mainly a review based on the WoS ranking, which may explain why it has been so widely cited over the past decade.

### Research hot spots and frontiers

It is well known that the study of frequently occurring keywords might reveal shifting patterns and major themes that are essential for understanding developments in the field. Therefore, keywords and burst keywords reflect the research hot spots and frontiers, as shown in **Figures 10**, **11**.

#### Hot spot 1: Risk factors and prognosis studies of HCC-MVI

This cluster is a larger system, which contains some keywords such as “survival”, “risk factor”, “prediction”, and “prognosis factor”. As research in the field of HCC-MVI has gained interest, abundant risk factors have been demonstrated to be strongly associated with the incidence and adverse outcome. A decade ago, several scholars described how to predict MVI and interpret its effects based on prognosis. The analysis of the results of previous documents revealed that two categories of risk factors can be identified for MVI: tumor-related features and tumor markers. The former included the tumor size, number, capsular, and satellitosis (17–19). The latter included alpha-fetoprotein (AFP), des-gamma-carboxy prothrombin (DCP), and inflammation-related indicators, such as platelet-to-lymphocyte ratio and neutrophil-to-lymphocyte ratio (17, 20, 21). The analysis showed enormous factors affecting MVI, and the prediction of MVI by a single factor may have a certain degree of deviation. Therefore, it is necessary to conduct clinical analysis by integrating multiple characteristics to improve the accuracy of preoperative prediction. Shirabe et al. (20) revealed that tumor size, serum DCP levels, and the maximum standardized uptake value (SUVmax) of 2-[18F]-fluoro-2-deoxy-D-glucose positron emission tomography were the independent clinical predictors of MVI after multivariate analyses. Meanwhile, the doctors and radiologists at the Centre for Imaging Science of Sungkyunkwan University also combined two or more of the imaging features, including arterial peritumoral enhancement, a non-smooth tumor margin, and peritumoral hypointensity at the hepatobiliary phase, as preoperative imaging biomarkers to predict MVI through gadoxetic acid–enhanced magnetic resonance imaging. These biomarkers are associated with early recurrence after curative resection of a single HCC (22). Zhao et al. (17) constructed a scoring system based on the AFP/gamma-glutamyl transpeptidase level, tumor diameter, and tumor number. Their results indicated that, when the score was greater than 3, the incidence rate of MVI increased by five times, whereas the overall survival (OS) rate decreased significantly. Although different parameters were evaluated as the potential predictors for MVI of HCC, with high diagnostic performances, the reliability and reproducibility of part of conclusions remain being questioned.

Burst keywords refer to those widely cited in articles within a particular period, which are considered another significant indicator of research hot spots or emerging trends. As shown in **Figure 11**, the trend in hot keywords and topics over time shows that research hot spots have undergone a transition from early studies on the risk factors and prognosis studies of MVI to later studies on imaging characteristics and transarterial chemoembolization (TACE) therapy studies.

#### Hot spot 2: Imaging characteristic studies of HCC-MVI

Recently, this category has been one of the research hot spots, which mainly refers to “image”, which represents one of the strongest citation bursts, and “radiomics”, which reflects the largest cluster in the cluster view and timeline view of co-cited references. An important issue affecting HCC prognosis is the absence of a highly reliable factor to predict MVI preoperatively. Thus, several image radiomics studies have been incorporated into the MVI report. Jiang et al. (23) developed predictive models using eXtreme Gradient Boosting and deep learning based on computerized tomography images to estimate MVI preoperatively. However, Li et al. (24) verified through the promising technique of magnetic resonance elastography–based shear strain mapping technique that the low interface shear strain identified at tumor-liver boundaries was highly correlated with the presence of MVI. Except that, scholars have evaluated the SUVmax of preoperative fluorine-18 fluorodeoxyglucose positron emission tomography/computed tomography (CT) and summarized the combination of the two risk factors (SUVmax and AFP-L3), providing a reliable assessment for predicting MVI, with the sensitivity and specificity rates of 88.9% and 82.4%, respectively (25). In addition to this finding, on the basis of a retrospective study, Xu et al. (26) developed a computational approach that integrated 15 clinical factors and 12 imaging features from contrast-enhanced CT, which demonstrated a good performance in estimating MVI and clinical outcomes. Overall, the radiological methods based on medical image data can provide quantifiable image features and can achieve high predictive performance in clinical practice.

#### Hot spot 3: Transarterial chemoembolization therapy of HCC-MVI

This cluster mainly includes “TACE”, “hepatectomy”, and “liver transplantation”. As one of the important non-surgical treatment methods for HCC, TACE can reduce tumor recurrence rates and increase the OS rates, especially in patient in advanced stages (2, 27). The controversial focus of these studies was to investigate whether postoperative-TACE (p-TACE) improved the prognosis of patients with MVI. Yang et al. (28) included 13 studies, in which 824 patients received p-TACE after hepatectomy, which significantly improved the OS and recurrence-free survival compared with postoperative conservative treatment in patients with HCC accompanied by MVI after curative resection. Similarly, Li et al. (29) considered p-TACE, a safe intervention, which reduced tumor recurrence rates and improved the OS for MVI using a high-quality meta-analysis. In contrast, the recent results showed that only patients with Barcelona Clinic Liver Cancer (BCLC) stage A, China Liver Cancer (CNLC) stage Ib, and American Joint Commission on Cancer (AJCC) stage II were found to benefit more from p-TACE, but not patients with BCLC stage 0/B, CNLC stage IIb, and AJCC stage IIIa (30). A part of studies declared that p-TACE was not inferior to more cycles when improving prognosis, strongly recommending only one cycle following R0 resection (30, 31). Moreover, Liu et al. (32) also confirmed that p-TACE was an independent protective factor in the subgroup of tumor ≤ 5 cm but not in the subgroup of tumor > 5. At present, the research suggested that MVI after resection is not a contraindication for liver transplantation (LT) and demonstrated that LT should be reconsidered in case of MVI and multilocular tumor occurrence (33). However, further studies with improved design are needed in this area.

Moreover, our study found that, as the hot spots of MVI research changed, so did the journals publishing MVI research. The journals have gradually shifted from *Hepatology* and *Journal of Hepatology* to *Frontiers in Oncology* and *Medicine*. This phenomenon of changing research hot spots may have a correlation with the specialties of the journals in which they have published.

### Managerial implications of the research

This research has tried to build a comprehensive network about HCC-MVI. We identified a continual increase in MVI studies from 2013 to 2022. The most upstanding country is China, followed by Korea and the US. At the same time, we noticed a strong cooperation between Chinese and American researchers in the domain. However, the influence and research quality of Chinese scholars in the field need to be further improved.

Furthermore, MVI is a poor prognosis factor for recurrence and long-term survival after hepatectomy or transplantation (3). Thus, the research of MVI and bibliometric analysis is beneficial for all relative clinicians and researchers. The study summarized the research hot spots and frontiers, which have undergone a transition from early studies on the risk factors and prognosis studies of MVI to later studies on imaging characteristics and TACE therapy studies. Therefore, the clinicians, researchers, and practitioners will develop an elaborate understanding of facilitating meaningful insight into future directions relative to advances in the MVI field.

### Research strengths and limitations

This study systematically evaluated the literature on HCC-MVI and provided a comprehensive and quantitative analysis of the most significant MVI articles, acknowledging the key contributions made to the evolution and advancement of this specialized field. However, we encountered several drawbacks. First, the data analyzed in the study were only from WoSCC-SCIE, and other databases, such as PubMed, Scopus, and Embase, were excluded, which could have resulted in the omission of relevant papers. Second, we only selected some documents (articles and reviews); excluded letters, books, and chapters; and defined the focus language as English, which may have caused some linguistic bias. Similarly, the multiple author identities of the author may bias some of the results of affiliated institutions. Third, the data generated from studies published in the current year (2022) were incomplete in our analysis. Therefore, because data are typically prone to frequent changes, our results only reflected the current state of HCC-MVI research. Fourth, the wealth of different countries should also be mentioned as a potential cause of research bias as this will limit investment in health research. Moreover, the size of a country’s population is another factor. Finally, self-citation is a significant problem that can influence these results.

## Conclusions

A systematic bibliometric analysis and visualization research is carried out to bridge the gap and to identify the hot spots in research. This manuscript aims to shed light on bibliometric analysis of MVI in HCC. Furthermore, it offers a groundwork for other HCC research studies. This bibliometric study identified a continual increase in MVI-related studies from 2013 to 2022, with China leading the field. China and the US became the most closely cooperated countries.

The citation analysis results revealed that the most productive and leading article on MVI of HCC is that by Bray F et al. (2018) (1) titled “Global cancer statistics 2018: Globocan estimates of incidence and mortality worldwide for 36 cancers in 185 countries” in “*CA-A Cancer Journal for Clinicians*”. The most prolific and dominant source is the “Frontiers in Oncology”, followed by the “Medicine”. Authors Bruix J and Lau Wan Yee have the maximum number of citations and articles published in the MVI field. Fudan University, China, emerges as the top most organization in this domain, followed by the Second Military Medical University.

“Survival” emerges as a dominating keyword, followed by “recurrence”. On the basis of the analysis of the temporal evolution of keywords, it is revealed that the research hot spots in the field have undergone a transition from early studies on the risk factors and prognosis studies of MVI to later studies on imaging characteristics and TACE therapy studies. This research manuscript is a boon for a researcher willing to work in the field of HCC-MVI domain. The network visualizations help to identify the research gaps termed “hot spots”. Future research can be conducted in these areas, which allows the researcher to minimize the time spent determining the exact topic and field of the study.

## Data availability statement

The original contributions presented in the study are included in the article/supplementary files. Further inquiries can be directed to the corresponding author.

## Author contributions

TH and JZ: article writing. TH, JZ, LX, TL, JL, and SY: performed image acquisition. TH, JZ, and KS: data collection. TH, JZ, SY, LX, TL, JL, JY, and GL: revised and improved the manuscript. All authors contributed to the article and approved the submitted version.
